# Very Successful

**Published:** 1884-07

**Authors:** 


VERY SUCCESSFUL.—Punch is responsible for the following; "First Country Doctor: Could you come to my place, Brown, to morrow morning? Second Country D.: All right, old man: what is it? First C. D.: Well, I've had a case of endocarditis, which I've very successfully treated with *convallaria majalis*, and I want your help with the *post-mortem*.

